# Potential Therapeutic Strategies for Intracranial Aneurysms Targeting Aneurysm Pathogenesis

**DOI:** 10.3389/fnins.2019.01238

**Published:** 2019-11-26

**Authors:** Zhao Liu, Kuerban Ajimu, Naibijiang Yalikun, Yongtao Zheng, Feng Xu

**Affiliations:** ^1^Department of Neurosurgery, Jingjiang People’s Hospital, Taizhou, China; ^2^Department of Neurosurgery, First People’s Hospital of Kashgar, Kashgar, China; ^3^Department of Neurosurgery, Hotan District People’s Hospital, Hotan, China; ^4^Department of Neurosurgery, Shanghai Medical College, Huashan Hospital Fudan University, Shanghai, China; ^5^Department of Neurosurgery, Kashgar Prefecture Second People’s Hospital, Kashgar, China

**Keywords:** intracranial aneurysms, rupture, therapeutic strategies, pathogenesis, inflammation

## Abstract

Subarachnoid hemorrhage resulting from intracranial aneurysms (IAs) is associated with high rates of morbidity and mortality. Although trigger mechanisms in the pathogenesis of IAs have not been fully elucidated, accumulating evidence has demonstrated that inflammation acts as a critical contributor to aneurysm pathogenesis. IAs is initiated by disruption and dysfunction of endothelial cells (ECs) caused by abnormal wall shear stress (WSS). Subsequently, vascular inflammation can trigger a series of biochemical reactions resulting in vascular smooth muscle cell (VSMC) apoptosis and migration, accompanied by inflammatory cell infiltration, secretion of various cytokines, and inflammatory factors. These changes result in degradation of vascular wall, leading to the progression and eventual rupture of IAs. Increasing our knowledge of the pathogenesis of these lesions will offer physicians new options for prevention and treatment. In this study, we review aneurysmal pathogenesis to seek for safe, effective, and non-invasive therapeutic strategies.

## Introduction

**FIGURE 1:**
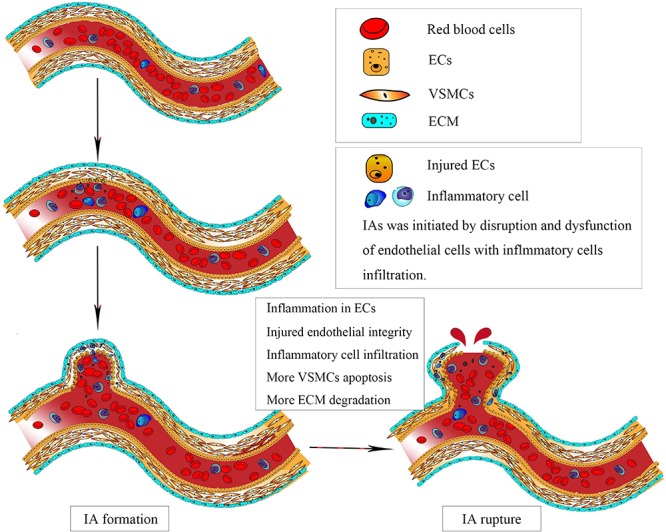
Intracranial aneurysm (IA) was initiated by disruption and dysfunction of endothelial cells (ECs) caused by abnormal physiological wall shear stress. Subsequently, vascular smooth muscle cells apoptosis and migration, accompanied by inflammatory cell infiltration, resulted in degradation of vascular wall, leading to the progression and eventual rupture of IAs.

Saccular intracranial aneurysms (IAs) are the most common cause of subarachnoid hemorrhage (SAH), which resulted in a high mortality and morbidity (Lawton and Vates, 2017). Approximately 3–5% of all IAs actually rupture, resulted in devastating SAH (Feigin V. L. et al., 2005). Mortality risks are between 30 and 45% for aneurysmal SAH, and up to 20% of survivors will be permanently disabled (Nieuwkamp et al., 2009). Although surgical clipping and endovascular therapy, including coiling alone and stent-assisting coiling, have been the main therapeutic methods for IAs, potentially serious complications related to those invasive procedures should not be neglected. Understanding the mechanisms underlying formation, progression, and rupture of IAs may help us to look for potential therapeutic strategy, especially safe and effective non-invasive therapies. Although the animal models were widely used to investigate the mechanism of aneurysm pathogenesis, those aneurysm samples were induced but not spontaneously formed. In addition, it is difficult to collect enough tissue for biochemical assays from human IAs; therefore, our understanding of the specific mechanism of IAs remains incompletely defined. Previous studies have demonstrated wall shear stress (WSS)-driven inflammation response of endothelial cells (ECs) was the initial step in the formation of IAs (Sho et al., 2002; Jamous et al., 2007). Subsequently, inflammatory cell infiltration, vascular smooth muscle cells (VSMCs) apoptosis and migration (Figure 1), and extracellular matrix (ECM) protein degradation promoted the progression and rupture of IAs (Hashimoto et al., 2006; Chalouhi et al., 2012; Signorelli et al., 2018). Therefore, agonists or inhibitors targeting different molecules involved in aneurysm formation and rupture may be potential therapeutic agents. In this review, we try to take aim at profiling the pathogenesis mechanism in formation and progression of IAs on the facet of cytology, which has positive impacts on deconstructing and deducing the potential therapeutic targets of IAs (Table 1).

**TABLE 1:** Targeted therapies for intracranial aneurysms (IAs).

**Pathway**	**Major targets**	**Mediators**
Endothelial dysfunction	PGE2-EP2 signaling	COX-2 inhibition Aspirin
	NF-κB	NF-κB p50 subunit
	MCP-1	MCP-1 inhibitor
	eNOS, iNOS	
	VCAM-1	HGF
	YAP	
Phenotypic modulation and Apoptotic of VSMCs	TNF-α	Infliximab
	KLF4	KLF4 inhibition
	MMPs	MMP inhibitor
	VCAM-1	HGF
	PPARs family	Pioglitazone
	IL-1β	
	microRNAs	MiR-370-3p, MiR-29b, MiR-9
Macrophages infiltration	MMPs	MMP inhibitor
	MCP-1	MCP-1 inhibitor
	PGE2 and EP2	S1P1 receptor agonist, EP2 antagonist
Lymphocytes and Mast cells	IL-1,3,4,6,8,13	
	MMP2 and MMP9	MMP inhibitor
	CD4+ T cell imbalance	
	TNF-β and TNF-α	Mast cell degranulation inhibitor

## Prevalence of Intracranial Aneurysms

According to various data, the prevalence of IAs in the general population ranges from 0.2% to 9.0% (Komotar et al., 2008; Chalouhi et al., 2011). Multiple IAs refer to the presence of two or more aneurysms of the intracranial arteries, which represent 7–15% of all the IAs (Kaminogo et al., 2003). IAs are more frequent in patients aged 35 to 60 years (Toth and Cerejo, 2018). In general, females are prone to be affected.

A generalization of the results of numerous studies demonstrates an average observed 1-year risk of aneurysm rupture of 1.4% and a 5-year risk of 3.4%, which is fatal in about 50% of the cases (Greving et al., 2014). A systematic review reported that the risks of aneurysm rupture per 100 patient-years were 0, 3.5, and 5.7% in patients aged 20–39, 40–59, and 60–79 years, respectively. At the same time, the risk of aneurysm rupture per 100 patient-years in female (2.6%, 95% CI, 1.8–3.6%) is higher than that of male (1.3%, 95% CI, 0.7–2.1%) (Rinkel et al., 1998).

## Risk Factors for Aneurysm Formation and Rupture

Identifying reliable indicators of risk for aneurysm formation and rupture can vastly improve clinical management of IAs. In the past few decades, researchers have identified several genetic factors, morphology parameters, and clinical conditions related to the growth and rupture of IAs. Significant evidence of linkage to IAs was found on chromosomes 13q and 8p22.2 (Santiago-Sim et al., 2009; Kim et al., 2011). In addition, the variants on chromosomes 8q and 9p are associated with IAs, which can be enhanced by cigarette smoking (Deka et al., 2010). Furthermore, the familiar occurrence suggested that genetic factors may be involved in the development of IAs.

The most ubiquitous parameter to evaluate the risk of aneurysm rupture is size. Although data from International Subarachnoid Aneurysm Trial (ISUIA) demonstrated that aneurysms less than 7 mm have a very low risk of rupture (Wiebers et al., 2003), several studies have shown that a large percentage of ruptured aneurysms are, in fact, smaller than 7 or even 5 mm (Froelich et al., 2016; Zheng et al., 2019). Aneurysm location is also an important factor in the risk of rupture. In a consecutive series of 1993 patients with saccular ruptured IAs, the three most common locations of ruptured IAs were the middle cerebral artery, anterior communicating artery, and middle cerebral artery (Korja et al., 2017). Additional morphology parameters including aspect ratio, size ratio, area ratio, and flow patterns were proposed to evaluate the risk of aneurysm rupture. However, most of these data were obtained from a single center, which cannot fully explain the factors affecting aneurysm rupture. Clinical factors related to IAs rupture include an old age, female, smoking, and aneurysm multiplicity (Feigin V. et al., 2005). There is some evidence that hypercholesterolemia and antithrombotic drugs may be protective with regard to IA formation and rupture (García-Rodríguez et al., 2013; Vlak et al., 2013).

## Inflammation and Intracranial Aneurysm

**FIGURE 2:**
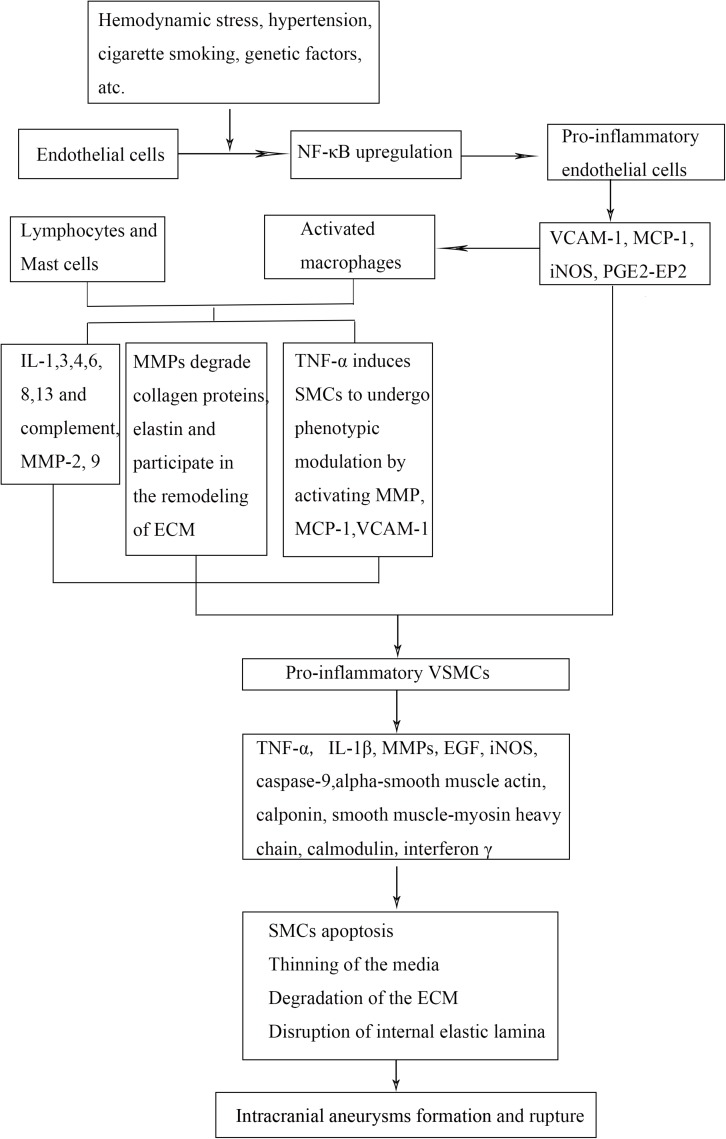
Mechanistic flow chart of IA pathobiology. Abnormal wall sheer stress on the endothelial cells (ECs) causes release of inflammatory mediators that recruit inflammatory cells into the damaged vessel walls. Smooth muscle cells undergo phenotypic modulation and apoptosis, which lead to breakdown of the surrounding extracellular matrix. These events lead to weakening of the arterial wall, aneurysm formation, and rupture. NF-κB indicates nuclear factor-κB; VCAM-1, vascular cell adhesion molecule 1; MCP-1, monocyte chemoattractant protein 1; iNOS, inducible nitric oxide synthase; IL, interleukin; TNF-α, tumor necrosis factor α; MMP, matrix metalloproteinase; PGE_2_–EP_2_, prostaglandin E_2_–E receptor 2; VSMCs, vascular smooth muscle cells; ECM, extracellular matrix; IA, intracranial aneurysm.

Under abnormal WSS, nuclear factor-κB (NF-κB) in cerebral arterial walls upregulates expression of downstream genes, such as monocyte chemoattractant protein 1 (*MCP-1*) and vascular cell adhesion molecule 1 (*VCAM-1*) gene, which lead to macrophage infiltration and endothelial dysfunction. Macrophages, via secretion of inflammatory cytokines and enzymes, recruit more inflammatory cells and induce SMC to undergo phenotypic modulation and apoptosis. Subsequently, the inflammatory response in vessel wall leads to disruption of internal elastic lamina, ECM degradation, and aneurysm formation. Persistent hemodynamic stress and inflammatory response lead to the progression and rupture of IA when the wall integrity is disrupted (Figure 2).

### Endothelial Cells

Intracranial aneurysms are vascular lesions characterized by the excessive degradation of ECM and chronic inflammation in the wall of arteries. Increasing evidence has shown that immunologic effect involved in aneurysm formation and rupture is related to ECs, VSMCs, and leukocytes. ECs can prevent luminal thrombosis though the barrier function between the vessel wall and the bloodstream. IAs are initiated by disruption and dysfunction of ECs caused by high WSS (Meng et al., 2007, 2014; Metaxa et al., 2010). Once abnormal WSS triggers prostaglandin E_2_–E receptor 2 (PGE_2_–EP_2_) signaling in endothelium, inflammatory pathway is amplified by NF-κB (Aoki et al., 2011). Therefore, the pathway of PGE_2_–EP_2_–NF-κB signaling maintains and continuously strengthens the inflammatory response, contributing to aneurysm formation.

NF-κB, a family of transcriptional factors, can activate the expression of some proinflammatory genes, such as *MCP-1* and *VCAM-1* genes, which lead to macrophage recruitment into the arterial wall and ECM degradation. Therefore, the effect of abnormal WSS on aneurysm formation is through the activation of acute and chronic inflammation in ECs, which results in endothelial dysfunction and weakening of endothelial integrity. ECs become elongated and realign with directional blood flow. The density or migration of ECs may change in response to the changes in the development of actin stress fibers. Both morphological and functional changes in ECs under abnormal hemodynamic stress alter the gene expression profile of ECs. Wang et al. (2009) demonstrated that extensive EC apoptosis is accompanied by reduction or absence of endothelial nitric oxide synthase (eNOS) expression. Decreased eNOS affects the biological activity of NO, a regulator of maintaining the stability of vascular tone, regulating the stability of blood pressure, and affecting the relaxation of smooth muscle. In addition, the damage of ECs induces the expression of inducible nitric oxide synthase (iNOS) in VSMCs and produces a large amount of nitric oxide free radicals, causing further damage to the vessel wall. Animal experiments have confirmed that iNOS is an important factor in the development of aneurysms. In iNOS gene knockout mice, the incidence of IAs is lower, and the apoptotic status of SMCs in aneurysms is reduced (Sadamasa et al., 2003).

Monocyte chemoattractant protein 1 secreted by ECs is another important step in aneurysm formation. It is generally believed that NF-κB upregulates the expression of MCP-1 in ECs by binding to two sites on the *MCP-1* gene. In addition, activation of MCP-1 is also affected by many other factors, such as various cytokines and shear stress. The expression of MCP-1 can cause macrophages and monocytes to infiltrate into the vascular wall. Also, the infiltrating cells can secrete MCP-1, leading to a self-amplification loop in the inflammatory environment, which causes the degradation of SMCs and ECM, further promoting the development of aneurysms. In the MCP-1 knockout mice, the expression of matrix metalloproteins and the incidence of aneurysm formation decreased significantly (Aoki et al., 2009). Loss of intact endothelium and inflammation infiltrating is the feature of aneurysm formation. Thus, targeting the endothelial barrier to prevent macrophage infiltration may be an effective and reasonable therapeutic strategy for IAs in the clinic in the future. It has been demonstrated that hepatocyte growth factor (HGF) concentrations were higher in IA sample and blood from patients with IAs, which protects against vascular inflammation (Peña-Silva et al., 2015). As HGF decreased the expression level of VCAM-1 and E-selectin in ECs, HGF signaling is a potential therapeutic strategy for IAs. Yes-associated protein (YAP) plays an important role in angiogenesis and vascular barrier maturation by regulating actin cytoskeleton remodeling and the metabolic activity of ECs. In animal experiments, endothelial-specific deletion of Yap/Taz led to aneurysm-like tip ECs and disrupted barrier integrity, which contributed to subsequent intracranial hemorrhage (Kim et al., 2017). Therefore, YAP in ECs may be a potential therapeutic site for neovascular diseases.

### Vascular Smooth Muscle Cells

Vascular smooth muscle cells, mainly concentrated in the media layer, produce ECM, which is the main component of the vessel wall. During the formation of IAs, VSMCs undergo proliferation and migration, apoptosis, and degeneration, accompanied by inflammatory cell infiltration and secretion of various cytokines and inflammatory factors. Structural and pathological changes in VSMCs play a key role in the progression and rupture of IAs. In response to ECs injury, VSMCs proliferate and migrate into the intimal layer, leading to myointimal hyperplasia. Subsequently, contractile (differentiated) VSMCs dedifferentiated into synthetic (dedifferentiated) VSMCs. Differentiated VSMCs are characterized by high levels of contractile gene expression and low ECM synthesis, whose physiological function is to regulate blood pressure and blood flow distribution (Nakajima et al., 2000; Kilic et al., 2005). The main marker of contractile VSMCs is alpha-smooth muscle actin, calponin, smooth muscle-myosin heavy chain, calmodulin, binding proteins, VSMC actin, etc. Dedifferentiated VSMCs have opposite functions to differentiated VSMCs, whose marker is osteopontin, epidermal growth factor (EGF), EGF family, epiregulin, etc. (Owens et al., 2004). Morphologically, spindle-like VSMCs change into spider-like cells and are sparsely arranged in aneurysm wall. The mechanism of phenotypic modulation of VSMCs in the pathogenesis of IAs is still poorly understood.

During the process of aneurysm formation, tumor necrosis factor-alpha (TNF-α) may play a pivotal role in the phenotypic regulation of SMCs. Specifically, TNF-α inhibited the contractile phenotype of SMCs as well as induced proinflammatory/matrix remodeling genes, such as matrix metalloproteinases (MMPs), VCAM-1, MCP-1, and interleukin 1β (IL-1β) (Jayaraman et al., 2005, 2008). The effect of TNF-α on phenotypic modulation of VSMCs is associated with increased expression of kruppel-like transcription factor 4 (KLF4), a known regulator of VSMC differentiation. Inhibition of KLF4 expression abrogated the expression of sputum-induced inflammatory genes and inhibited contractile genes (Ali et al., 2013). A series of *in vitro* and *in vivo* studies demonstrated the major members of peroxisome proliferator-activated receptor (PPAR) family, PPARγ and PPARβ/δ, are characterized by modulation of the vascular cell proliferation and vascular inflammation (Shimada et al., 2015). In cultured cerebral VSMCs, PPARβ/δ partially inhibited the phenotypic switch of brain VSMC by activating PI3K/AKT pathway, which regulates vascular remodeling in cerebral aneurysms. A study by the Hasan group found that inhibition of PPARγ function in VSMCs increased incidence and rupture rate of cerebral aneurysms, which upregulated the gene expression of TNF-α, MCP-1, Cxcl1, MMP-3, and MMP-9 (Shimada et al., 2015). The main role of MMPs is to degrade collagen proteins, elastin, and non-collagen glycoproteins and to participate in the remodeling of ECM. Under normal static conditions, the expression of MMPs is very limited, which are present as inactive zymogens. In pathological states, MMPs are secreted by VSMCs in response to inflammatory response factors, such as NF-κB, TNF-α, IL-1β, and free radicals. Through the mechanisms of “zinc-cysteine theory,” MMPs are activated and exert biological effects in the formation and rupture of IAs. In general, phenotypic regulation of VSMCs in the aneurysm wall is closely related to the remodeling of aneurysm walls and the mechanism of aneurysm rupture.

The balance between apoptosis and production of VSMCs is fundamental to development and remodeling of normal vessel wall; however, excessive apoptosis leads to vascular-related diseases, such as IAs. Previous studies have demonstrated apoptosis of medial SMCs in the formation of saccular cerebral aneurysms (Kondo et al., 1998; Guo et al., 2007). Furthermore, loss and apoptosis of SMCs play an important role not only in the development of IAs but also in the rupture of aneurysm (Sakaki et al., 1997). In the pathogenesis of IAs, two major causes of VSMCs apoptosis are hemodynamic stimulation and inflammation. *In vitro* experiments demonstrated that elevated mechanical stress can induce apoptosis of cultured VSMCs within the media (Sedding et al., 2008). Cyclic tensile force can upregulate p53 protein expression and increase transcriptional activity, which resulted in increased apoptosis of VSMCs. At the same time, mechanical stress increases calpain activity, which counteracts excessive VSMCs apoptosis though degrading p53. Reversely, inhibition of calpain activation increases p53 expression, leading to a further increase of apoptotic rate of VSMCs (Sedding et al., 2008). Additionally, flow-dependent No release inhibits the proliferation of VSMCs and may initiate apoptosis by activation of caspase 3 (Penn et al., 2014; Soldozy et al., 2019). Inflammation cytokines, such as IL-1β, interferon γ, and iNOS, also contribute to VSMC apoptosis. Moriwaki et al. (2006) reported IL-1β was detected in vascular media at an early stage of IA formation in an animal model. Compared with wild-type mice, the number of apoptotic cells was significantly reduced and caspase-1 expression increased in IL-1β−/− mice. Similarly, Sadamasa et al. (2003) reported that the number of apoptotic VSMCs as well as the size of experimental IAs in the iNOS+/+group, compared with the iNOS−/− group, were significantly greater. Inflammatory responses leading to VSMC apoptosis can also be initiated by oxidative stress via the intrinsic pathway (Laaksamo et al., 2013). Therefore, regulation of iNOS, IL-1β, or caspase-9 may be a potential therapeutic target in the prevention of the progression of IAs.

Currently, more and more research focuses on the influence of microRNAs (miRNAs) on the formation and development of IAs. MiRNA, a short and single-stranded non-coding RNA, consists of 22 nucleotides. As a group of non-coding RNAs, miRNAs can negatively regulate expression of genes by binding to the 3’-UTR region of target mRNAs, thereby inducing degradation of mRNAs and inhibiting repression of protein synthesis. Prior studies have demonstrated that miRNAs can regulate the proliferation, migration, and apoptosis of VSMCs. Hou et al. (2017) reported that miRNA-370-3p was involved in the development of IAs by inhibiting VSMC proliferation via targeting KDR/AKT signaling. Similarly, aberrant expression of miR-29b and miRNA-9 contributes to the development and rupture of IAs by phenotypic modulation and suppressing proliferation of VSMCs (Luo et al., 2016; Sun et al., 2017). These studies provide theoretical basis for further investigation of potential clinical prevention and treatment of IAs via VSMC approach.

### Leukocytes

Inflammatory response that follows hemodynamic endothelial injury is the basic step in the pathogenesis of IAs (Jamous et al., 2007). The infiltration of macrophages, a subset of leukocytes, is commonly observed during the progression of IAs (Chyatte et al., 1999; Kataoka et al., 1999; Frösen et al., 2004). Inhibiting macrophage recruitment and accumulation in the IA wall through pharmacological depletion of macrophages can remarkably decrease the incidence and size of IAs in animal models (Kanematsu et al., 2011). As two major subtypes of human macrophages, the M1 and M2 cells present extremely different functions and sometimes even antagonized each other. M1 macrophages are proinflammatory cells, whereas M2 macrophages are involved in inflammation resolution and tissue repair (Mantovani et al., 2005; Hasan et al., 2012). An imbalance of macrophage M1–M2 polarization is often associated with inflammatory conditions. M1 macrophages play a key role in vascular remodeling by releasing MMPs, in particular MMP-2 and MMP-9 (Aoki et al., 2007a). In addition, inhibited MMP-2 and MMP-9 expression and decreased macrophage recruitment in MCP-1 knockout mice accompanied with a remarkably reduced incidence of IAs and inflammatory response (Aoki et al., 2009; Kanematsu et al., 2011). Aoki et al. (2017) proposed that PGE_2_–EP_2_–NF-κB signaling cascade in macrophages was a potential therapeutic target for IAs. NF-κB activation can be detected in infiltrating macrophages in IA lesions (Aoki et al., 2007b). Specific inhibition of E_2_(PGE_2_)–EP_2_–NF-κB signaling in macrophages can inhibit macrophage infiltration and expression of proinflammatory factors in the IA wall during the process of IA formation. Oral administration of PF-04418948, a selective EP_2_ antagonist, can reduce the size of induced IAs, and furthermore, it reduced the thinning and dilating of vessel walls without significantly affecting systemic blood pressure (Aoki et al., 2017). Therefore, the potential therapeutic target of IAs should cover the macrophage and other elements interrelating the macrophage infiltration or involving the inflammatory response of the macrophage. Recently, Yamamoto et al. (2017) proposed that a selective sphingosine-1-phosphate receptor type 1(S1P1 receptor) agonist, ASP4058, can be used as a candidate for treating IAs. They identified that S1P1 was present on the endothelium, which can promote barrier function of endothelium. In an *in vitro* experiment, ASP4058 significantly suppressed the migration of macrophages across an endothelial monolayer and promoted endothelial integrity. They further confirmed in animal experiments that oral administration of ASP4058 significantly reduced the size of the IAs though decreasing the vascular permeability and macrophage infiltration (Yamamoto et al., 2017).

Although immunohistochemistry of IAs wall and peripheral blood in IAs patients revealed the presence of lymphocytes in lesions suggesting involvement of this type of cell in the pathogenesis, it remains unclear whether lymphocytes directly participate in progression and rupture of IAs. Lymphocyte-deficient and wild-type mice were used to examine the contribution of lymphocytes in a model of IA. Lymphocyte-deficient group showed fewer IA formations and ruptures than did the wild-type group. Although macrophage infiltration showed no differences in two groups, there were significant differences in IL-6, MMP-2, MMP-9, and smooth muscle myosin heavy chain between the groups. Therefore, we suspect that lymphocytes participate in the formation of aneurysms by degrading and remodeling the aneurysm wall (Sawyer et al., 2016). However, Miyata et al. (2017) reported that although T cells are detectable in the wall of IAs, they failed to affect the degenerative changes of arterial walls, macrophage infiltration, and the formation and progression of IAs. Furthermore, the studies on the peripheral blood of the IAs patients demonstrated the abnormal proportion of CD4+ T cells and a succession of accompanying tremendous unbalanced features, such as the expression disorders of T helper-1, T helper-17, and the T helper-2 and regulatory T activities, which was adjusted by the increase of IFN-g, TNF-a, and IL-17 production and the decrease of IL-10 production from total CD4+ T cells (Zhang et al., 2016). The imbalance of CD4+ T cell subset might exacerbate the disease through a positive feedback loop, leading to a higher state of inflammation in IAs. Therefore, we need more clinical and basic experiments to explore the role of lymphocytes in the formation and rupture of aneurysms. In addition, we need to subdivide lymphocytes to explore the mechanism of their effects on IAs.

Mast cells are important proinflammatory cells involved in various vascular diseases through the release of prostaglandins and leukotrienes. Then, a variety of proinflammatory cytokines were secreted slowly after mast cells were activated, including TNF-α, IL-1, IL-3, IL-4, IL-6, IL-8, IL-13, and transforming growth factor-beta (TGF-β). In Eliisa’s study, the samples were resected intraoperatively from 16 unruptured and 20 ruptured saccular IAs. Mast cells were found in 9 of 36 aneurysms, and all those aneurysms showed a damaged luminal endothelium (Ollikainen et al., 2014). The presence of mast cells was associated with a larger of CD3+ T lymphocytes and CD68+ macrophage. Thus, mast cells might be involved in the regulation of inflammatory responses in the wall of IAs, together with other inflammatory cells. By releasing the serine proteases, such as chymases and tryptases, which leads to the activation of MMP-2 and MMP-9, mast cells degrade pericellular matrices, which may result in VSMC apoptosis and detachment of ECs. Moreover, mast cells are also powerful angiogenic cells, which may promote the formation of neovessels in the aneurysm wall. In particular, iron deposits were found in aneurysm wall containing mast cells and neovessels, indicating newly formed endothelium damaged by microhemorrhages. High neovessel density and inflammatory cells infiltration also showed evidence of degeneration of IAs wall. Due to the important role of mast cells in the formation of aneurysms, inhibitors of mast cells may be a novel potential therapeutic strategy for IAs. Inhibitors of mast cell degranulation can effectively decrease the size of IAs in animal experiments, through the inhibition of chronic inflammation and macrophage infiltration, and decreasing the expression of MMPs and IL-1β (Ishibashi et al., 2010).

## Potential Therapeutic Approaches

In the accumulation of knowledge about the involvement of inflammatory process in IA formation, progression, and rupture, the candidate drugs interfere with inflammatory response have potential importance. One of the promising drugs among these agents is aspirin through its inhibitory effect on COX-2 and microsomal prostaglandin E2 synthase-1 (mPGES-1). In Hasan’s study, the expression of COX-2, mPGES-1, and inflammatory cells in IA walls from patients who were given aspirin (81 mg daily) for 3 months decreased significantly (Hasan et al., 2013). Furthermore, the expression of some inflammatory molecules involved in IAs pathology, such as MMP-9 and MCP-1, decreased in mice treated with aspirin or COX-2 inhibitor. Another anti-inflammatory agent is atorvastatin, which could be a beneficial therapy to prevent aneurysm rupture. In animal models, atorvastatin can inhibit the expression of VCAM-1, E-selectin, and P-selectin; downregulate thrombomodulin and cholesterol-1; and inhibit the activation of IL-1, IL-6, TNF-α, and MMP-2. Additionally, atorvastatin can attenuate the progression in cerebral aneurysms by promoting angiogenesis and vascular repair (Aoki et al., 2008; Li et al., 2014). Although the exact mechanism that these hormones act on the inflammatory cascade was not fully understood, studies have shown that administration of sex hormones can lower the risk of aneurysm formation and rupture. Evidence obtained from animal studies suggests that estrogen suppresses inflammatory signaling through the inhibition of NF-κB and protects against oxidative stress in CNS, which could reduce the frequency of aneurysm formation (Bruce-Keller et al., 2000; Wunderle et al., 2014).

Given the critical role of macrophages in the etiology of aneurysm formation and rupture, medicine targeting macrophage are likely to represent novel strategies for IA treatment. One of the promising drugs among these agents is PPARγ activation through their potent induction on alternative M2 phenotype. At the same time, reduced infiltration of M1 macrophage is observed in mice model treated with pioglitazone, suggesting that PPARγ may be a potential target for preventing IA rupture (Bouhlel et al., 2007; Shimada et al., 2015). Additionally, the other candidate drugs include some protease inhibitors including MMP inhibitors, free radical scavengers, and Ca^2+^ channel blockers. In recent years, although animal experiments confirmed drugs is a promising therapy for IAs, a focused and sustained research effort will be necessary to be applied in clinical practice.

## Conclusion

Animal models and clinical studies have shown that vascular remodeling and inflammatory cascades were the important mechanism for IA formation, progression, and rupture. In this process, there are three types of cells that play a crucial role, ECs, VSMCs, and leukocytes, including macrophages, lymphocytes, and mast cells. Abnormal WSS leads to EC damage, which initializes the inflammatory response, in which PGE_2_–EP_2_–NF-κB signal plays a significant role. In response to EC injury, structural and pathological changes in SMCs play a pivotal role in the progression and rupture of IAs, accompanied by inflammatory cell infiltration and secretion of various cytokines and inflammatory factors. Finally, with the participation of various types of inflammatory cells, degeneration of the blood vessel wall contributed to the formation of IAs. Therefore, a multitude of studies targeting the above cells have been investigated in animal models with promising results, which may be safe and effective non-invasive therapeutic strategies.

## Author Contributions

Conception and design: YZ and FX; Drafted the article: ZL and KA. All authors listed have made a substantial, direct and intellectual contribution to the work, and approved it for publication.

## Conflict of Interest

The authors declare that the research was conducted in the absence of any commercial or financial relationships that could be construed as a potential conflict of interest.
